# Theorems and Application of Local Activity of CNN with Five State Variables and One Port

**DOI:** 10.1155/2012/674243

**Published:** 2012-04-12

**Authors:** Gang Xiong, Xisong Dong, Li Xie, Thomas Yang

**Affiliations:** ^1^State Key Laboratory of Management and Control for Complex Systems, Institute of Automation, Chinese Academy of Sciences, Beijing 100190, China; ^2^Department of Information Science and Electronics Engineering, Zhejiang University, Hangzhou 310027, China; ^3^The Department of Electrical, Computer, Software, and Systems Engineering, Embry-Riddle Aeronautical University, Daytona Beach, FL 32114, USA

## Abstract

Coupled nonlinear dynamical systems have been widely studied recently. However, the dynamical properties of these systems are difficult to deal with. The local activity of cellular neural network (CNN) has provided a powerful tool for studying the emergence of complex patterns in a homogeneous lattice, which is composed of coupled cells. In this paper, the analytical criteria for the local activity in reaction-diffusion CNN with five state variables and one port are presented, which consists of four theorems, including a serial of inequalities involving CNN parameters. These theorems can be used for calculating the bifurcation diagram to determine or analyze the emergence of complex dynamic patterns, such as chaos. As a case study, a reaction-diffusion CNN of hepatitis B Virus (HBV) mutation-selection model is analyzed and simulated, the bifurcation diagram is calculated. Using the diagram, numerical simulations of this CNN model provide reasonable explanations of complex mutant phenomena during therapy. Therefore, it is demonstrated that the local activity of CNN provides a practical tool for the complex dynamics study of some coupled nonlinear systems.

## 1. Introduction

Coupled nonlinear dynamical systems have been widely studied in recent years. However, the dynamical properties of these systems are difficult to deal with. Although the research on emergence and complexity has gained much attention during the past decades, the determination, prediction, and control of the complex patterns generated from high-dimensional coupled nonlinear systems are still far from perfect. Nature abounds with complex patterns and structures emerging from homogeneous media, and the local activity is the origin of these complexities [1, 2]. The cellular neural network (CNN), firstly introduced by Chua and Yang [3] as an implementable alternative to fully connected Hopfield neural network, has been widely studied for image processing, robotic, biological versions, and higher brain functions, and so on [3]. Many of the coupled nonlinear systems can be modeled and studied via the CNN paradigm [4]. The local activity proposed by Chua asserts that a wide spectrum of complex behaviors may exist if the cell parameters of the corresponding CNN are chosen in or nearby the edge of chaos [2, 4]. There have been quite a few new methods developed for complex systems [5–8], and local activity has attracted the attention of many researchers. Now, local activity has been successfully applied to the research of complex patterns generated from several CNNs in physical, biological, and chemical domains, such as Fitzhugh-Nagumo equation [9], Brusselator equation [10], Gierer-Meinhart equation [11], Oregonator equation [12], Hodgkin-Huxley equation [13], Van Der Pol equation [14], the biochemical model [15], coupled excitable cell model [16], tumor growth and immune model [17], Lorenz model [18], advanced image processing [19], Rossler equation [20], images analysis [21, 22], data prediction [23], neutron transport equation [24], vision safety [25], retinomorphic model [26], and theory research [27–30], and so forth.

Although Chua presents the main theorem of local activity at a cell equilibrium point [1, 2], it is actually difficult to “test” directly the complex patterns of the high-dimensional coupled nonlinear systems, since the theorem contains no recipe for finding whether a variable actually exists or not. It is necessary to develop some mathematical criteria according to the numbers of the variables and ports; that is the topic addressed in this paper.

The remaining of this paper is organized as follows. The local activity of CNN is introduced in Section 2. A set of theorems for testing the local activity of reaction-diffusion CNN with five state variables and one port are set up in Section 3. As an application of the theorems, a coupled reaction-diffusion CNN of hepatitis B Virus (HBV) mutation-selection model is introduced, aiming at describing HBV mutation in the therapeutic process. The bifurcation diagrams of this CNN are developed and some numerical simulations are presented in Section 4. Concluding remarks are given in Section 5.

## 2. Local Activity Theory of CNN

The CNN architecture is composed of a two-dimensional *M* × *N* array of cells. Each cell is denoted by *C*(*i*, *j*), where *i* = 1,2,…, *M*, *j* = 1,2,…, *N*. The dynamics of each cell is given by the equation:

(1) $$\begin{array}{c} {\dot{x}}_{ij}=-x_{ij}+\overset{r}{\underset{k=-r}{\sum }}\overset{r}{\underset{l=-r}{\sum }}a_{kl}y_{i+k\;j+l}+\overset{r}{\underset{k=-r}{\sum }}\overset{r}{\underset{l=-r}{\sum }}b_{kl}u_{i+k\;j+l}+z_{ij}, \end{array}$$

where *x*_*ij*_, *y*_*ij*_, *u*_*ij*_ are the state, output, and input variables of the cell, respectively. *a*_*k*,*l*_, *b*_*k*,*l*_, *z*_*ij*_ are the elements of the A-template, the B-template, and threshold, respectively. *r* is the radius of influence sphere. The output *y*_*ij*_ is the piece-wise linear function given by

(2) $$\begin{array}{cc} & y_{i,j}=\frac{1}{2}\left(\left|x_{i,j}+1\right|-\left|x_{i,j}-1\right|\right),\\ & \;i=1,2,\ldots ,M;\;j=1,2,\ldots ,N. \end{array}$$

Clearly, CNN with different template elements may have different functions.

A vast majority of active homogeneous media that are known to exhibit complexity are modeled by a reaction-diffusion partial differential equation (PDE):

(3) $$\begin{array}{c} \frac{\partial x_{i}}{\partial t}=f_{i}\left(X\right)+D_{i}\left(\frac{\partial^{2}x_{i}}{\partial x^{2}}+\frac{\partial^{2}x_{i}}{\partial y^{2}}+\frac{\partial^{2}x_{i}}{\partial z^{2}}\right),\;\;i=1,2,\ldots ,n, \end{array}$$

where *X* = (*x*_1_, *x*_2_,…*x*_*n*_) is state variables, (*x*, *y*, *z*) is spatial coordinates, *f*_*i*_(*x*_1_, *x*_2_,…, *x*_*n*_) is a coupled nonlinear vector function called the kinetic term, and *D*_1_, *D*_2_,…, *D*_*n*_ are constants called diffusion coefficients. Replacing the Laplace in above formulation by its discrete version yields

(4) $$\begin{array}{c} \frac{\partial^{2}x_{i}}{\partial x^{2}}+\frac{\partial^{2}x_{i}}{\partial y^{2}}+\frac{\partial^{2}x_{i}}{\partial z^{2}}\to \nabla^{2}X_{\alpha ,\beta ,\gamma }, \end{array}$$

where

(5) $$\begin{array}{cc} & {\left(\nabla^{2}X_{\alpha ,\beta ,\gamma }\right)}_{i}\\ & \;\;=x_{i}\left(\alpha -1,\beta ,\gamma \right)+x_{i}\left(\alpha +1,\beta ,\gamma \right)+x_{i}\left(\alpha ,\beta -1,\gamma \right)\\ & \;\;\;+x_{i}\left(\alpha ,\beta +1,\gamma \right)+x_{i}\left(\alpha ,\beta ,\gamma -1\right)\\ & \;\;\;+x_{i}\left(\alpha ,\beta ,\gamma +1\right)-6x_{i}\left(\alpha ,\beta ,\gamma \right). \end{array}$$

Chua et al. have introduced reaction-diffusion CNN equations:

(6) $${\dot{X}}_{\alpha ,\beta ,\gamma }=f\left(X_{\alpha ,\beta ,\gamma }\right)+D\nabla^{2}X_{\alpha ,\beta ,\gamma },\;$$

where *D* = diag⁡(*D*_1_, *D*_2_,…*D*_*n*_), ${\dot{X}}_{\alpha ,\beta ,\gamma }$ denotes the state variable located at a point in three-dimensional space with spatial coordinates. Chua refers to the process of transforming a PDE into a reaction-diffusion CNN [2].

From Chua and his collaborators' point, PDEs are merely mathematical abstractions of nature, and the concept of a continuum is in fact an idealization of reality. Even the collection of all electrons in a solid does not form a continuum, because much volume separating the electrons from the nucleus represents a vast empty space [2]. Reaction-diffusion CNNs have been used to model some phenomena with important practical backgrounds, which were described by PDEs.

Generally speaking, in a reaction-diffusion CNN, every cell has *n* state variables, but only *m* (*m* ≤ *n*) state variables couple directly to their nearest neighbors via “reaction-diffusion”. Consequently, each cell has the following state equations:

(7) $$\begin{array}{c} {\dot{V}}_{a}=f_{a}\left(V_{a},V_{b}\right)+I_{a},\\ {\dot{V}}_{b}=f_{a}\left(V_{a},V_{b}\right), \end{array}$$

where

(8) $$\begin{array}{cc} V_{a} & ={\left[V_{1},V_{2},\ldots ,V_{m}\right]}^{\text{T}},\;V_{b}={\left[V_{m+1},V_{m+2},\ldots ,V_{n}\right]}^{\text{T}},\;\\ f_{a} & ={\left[f_{1}\left(V_{a},V_{b}\right),f_{2}\left(V_{a},V_{b}\right),\ldots ,f_{m}\left(V_{a},V_{b}\right)\right]}^{\text{T}},\\ f_{b} & ={\left[f_{m+1}\left(V_{a},V_{b}\right),f_{m+2}\left(V_{a},V_{b}\right),\ldots ,f_{n}\left(V_{a},V_{b}\right)\right]}^{\text{T}},\;\\ I_{a} & ={\left[D_{1}\nabla^{2}V_{1},D_{2}\nabla^{2}V_{2},\ldots ,D_{m}\nabla^{2}V_{m}\right]}^{\text{T}}. \end{array}$$

The cell equilibrium point *Q*_*i*_ = (*V*_*a*_^*i*^, *V*_*b*_^*i*^)(∈R^*n*^) of equation (7) can be determined via

(9) $$\begin{array}{c} f_{a}\left(V_{a},V_{b}^{}\right)=0,\\ f_{b}\left(V_{a},V_{b}^{}\right)=0. \end{array}$$

The Jacobian matrix at the equilibrium point *Q*_*i*_ has the following form:

(10) $$J\left(Q_{i}\right)={\left[a_{kl}\left(Q_{i}\right)\right]}_{n\times n}=\left[\begin{array}{cc} {\text{A}}_{aa}\left(Q_{i}\right) & {\text{A}}_{ab}\left(Q_{i}\right)\\ {\text{A}}_{ba}\left(Q_{i}\right) & {\text{A}}_{bb}\left(Q_{i}\right) \end{array}\right],$$

where A_*kl*_(*Q*_*i*_) are called cell parameters and

(11) $$\begin{array}{c} {\text{A}}_{aa}\left(Q_{i}\right)=\left[\begin{array}{ccc} \frac{\partial f_{1}}{\partial V_{1}} & \cdots & \frac{\partial f_{1}}{\partial V_{m}}\\ \vdots & \ddots & \vdots \\ \frac{\partial f_{m}}{\partial x_{1}} & \cdots & \frac{\partial f_{m}}{\partial x_{m}} \end{array}\right],\\ {\text{A}}_{ab}\left(Q_{i}\right)=\left[\begin{array}{ccc} \frac{\partial f_{1}}{\partial V_{m+1}} & \cdots & \frac{\partial f_{1}}{\partial V_{n}}\\ \vdots & \ddots & \vdots \\ \frac{\partial f_{m}}{\partial x_{m+1}} & \cdots & \frac{\partial f_{m}}{\partial x_{n}} \end{array}\right],\\ {\text{A}}_{ba}\left(Q_{i}\right)=\left[\begin{array}{ccc} \frac{\partial f_{m+1}}{\partial V_{1}} & \cdots & \frac{\partial f_{m+1}}{\partial V_{m}}\\ \vdots & \ddots & \vdots \\ \frac{\partial f_{n}}{\partial x_{1}} & \cdots & \frac{\partial f_{n}}{\partial x_{m}} \end{array}\right],\;\\ {\text{A}}_{bb}\left(Q_{i}\right)=\left[\begin{array}{ccc} \frac{\partial f_{m+1}}{\partial V_{m+1}} & \cdots & \frac{\partial f_{m+1}}{\partial V_{n}}\\ \vdots & \ddots & \vdots \\ \frac{\partial f_{n}}{\partial x_{m+1}} & \cdots & \frac{\partial f_{n}}{\partial x_{n}} \end{array}\right]. \end{array}$$

The local state equations at the cell equilibrium point *Q*_*i*_ are defined via

(12) $$\begin{array}{c} {\dot{V}}_{a}={\text{A}}_{aa}V_{a}+{\text{A}}_{ab}V_{b}^{}+I_{a},\\ {\dot{V}}_{b}={\text{A}}_{ba}V_{a}+{\text{A}}_{bb}V_{b}^{}. \end{array}$$

**Definition 1**

(13) $$Y_{Q}\left(s\right)=sI-{\text{A}}_{aa}-{\text{A}}_{ab}{\left(sI-{\text{A}}_{bb}\right)}^{-1}{\text{A}}_{ba}$$

is called the admittance matrix at the cell equilibrium point *Q*_*i*_.

**Lemma 2**

A reaction-diffusion CNN cell is called locally active at the equilibrium point *Q*_*i*_ if and only if, its admittance matrix at *Q*_*i*_ satisfies at least one of the following four conditions [4].

1. *Y*_*Q*_(*s*) has a pole in *Re*[*s*] > 0.
2. $Y_{Q}^{H}(i\omega )={\bar{Y}}_{Q}(i\omega )+Y_{Q}(i\omega )<0$ for some *ω* = *ω*_0_, where *ω*_0_ is any real number.
3. *Y*_*Q*_(*s*) has a simple pole *s* = *iω*_*p*_ on the imaginary axis, where its associated residue matrix:
  (14) $$k_{1}=\{\begin{array}{cc} \underset{s\to i\omega_{p}}{\lim}\left(s-i\omega_{p}\right)Y_{Q}\left(s\right), & \text{if}\;\omega_{p}<\infty \\ \underset{\omega_{p}\to \infty }{\lim}\frac{Y_{Q}\left(i\omega_{p}\right)}{i\omega_{p}}, & \text{if}\;\omega_{p}=\infty \end{array}$$
  is either a complex number or a negative real number.
4. *Y*_*Q*_(*s*) has a multiple pole on the imaginary axis.

**Definition 3**

The cell equilibrium point *Q*_*i*_ is called stable if and only if, all the real parts of eigenvalue *λ*_*i*_ of *Jacobian *matrix at the equilibrium point *Q*_*i*_ are negative [2].

**Definition 4**

A “reaction-diffusion” CNN with *n* state variables and *m* ports is said to be operating on the “edge of chaos” with respect to an equilibrium point *Q*_*i*_ if and only if, *Q*_*i*_ is both locally active and stable when *I*_*a*_ = 0 [4].

Using the above lemma and definitions, the bifurcation of CNN with respect to an equilibrium point can be divided into three parts: the edge of chaos domains (the locally active and stable domains), the locally active and unstable domains, and the locally passive domains. Numerical simulations indicated that many complex dynamical behaviors, such as oscillatory patterns, chaotic patterns, or divergent patterns, may emerge if the selected cell parameters are located in or nearby the edge of chaos domains.

## 3. Analytical Criteria for Local Activity of CNN with Five State Variables and One Port

For the reaction-diffusion CNN with five state variables and one port, its local state equations have the form

(15) $$\begin{array}{c} {\dot{V}}_{a}={\text{A}}_{aa}V_{a}+{\text{A}}_{ab}V_{b}+I_{a},\\ {\dot{V}}_{b}={\text{A}}_{ba}V_{a}+{\text{A}}_{bb}V_{b}, \end{array}$$

where

(16) $$\begin{array}{c} V_{a}=\left[V_{1}\right],\;V_{b}={\left[\begin{array}{cccc} V_{2} & V_{3} & V_{4} & V_{5} \end{array}\right]}^{T},\;I_{a}=\left[I_{1}\right],\\ {\text{A}}_{aa}=\left[a_{11}\right],\;{\text{A}}_{ab}=\left[\begin{array}{cccc} a_{12} & a_{13} & a_{14} & a_{15} \end{array}\right],\\ {\text{A}}_{ba}={\left[\begin{array}{cccc} a_{21} & a_{31} & a_{41} & a_{51} \end{array}\right]}^{T},\\ {\text{A}}_{ba}=\left[\begin{array}{c} a_{21}\\ a_{31}\\ a_{41}\\ a_{51} \end{array}\right],\;{\text{A}}_{bb}=\left[\begin{array}{cccc} a_{22} & a_{23} & a_{24} & a_{25}\\ a_{32} & a_{33} & a_{34} & a_{35}\\ a_{42} & a_{43} & a_{44} & a_{45}\\ a_{52} & a_{53} & a_{54} & a_{55} \end{array}\right]. \end{array}$$

The corresponding CNN cell admittance matrix *Y*_*Q*_(*s*) is given by [1].

(17) $$\begin{array}{cc} Y_{Q}\left(s\right) & =sI-{\text{A}}_{aa}-{\text{A}}_{ab}{\left(s\text{I}-{\text{A}}_{bb}\right)}^{-1}{\text{A}}_{ba}\\ & =s-a_{11}-\frac{T_{1}s^{3}+K_{1}s^{2}+L_{1}s+\Delta_{1}}{s^{4}+Ts^{3}+Ks^{2}+Ls+\Delta }, \end{array}$$

where *T*, *T*_1_, *K*, *K*_1_, *L*, *L*_1_, Δ, Δ_1_ are the parameters of *a*_*ij*_'s.

**Theorem 5**

A necessary and sufficient condition for *Y*_*Q*_(*s*) to satisfy condition (1) in Lemma 2 is that ∃*s*, such that *g*(*s*) = 0  (*Re*[*s*] > 0), and any one of the following conditions holds.

1. *f*(*s*) ≠ 0.
2. *f*(*s*) = 0, and *m* > *n*, where *s* is *m* and *n* orders zero point of *g*(*s*) and *f*(*s*), respectively, where *f*(*s*) = *T*_1_ *s* ^3^ + *K*_1_ *s* ^2^ + *L*_1_ *s* + Δ_1_, *g*(*s*) = *s* ^4^ + *Ts* ^3^ + *Ks* ^2^ + *Ls* + Δ.

**Proof**

Obviously proved.

Denote

(18) $$\begin{array}{cc} & E=-a_{11},\;F=-TT_{1}+K_{1}-a_{11}\left(-2K+T^{2}\right),\\ & P=LL_{1}-K\Delta_{1}-\Delta K_{1},\\ & Q=\Delta_{1}+KK_{1}-TL_{1}-LT_{1},\\ & G=-a_{11}\left(2\Delta +K^{2}-2LT\right)-Q,\\ & H=-a_{11}\left(L^{2}-2\Delta K\right)-P,\\ & I=-a_{11}\Delta^{2}-\Delta \Delta_{1},\\ & g\left(Q\right)=EQ^{4}+FQ^{3}+GQ^{2}+HQ+I,\\ & h\left(\lambda \right)=-\left(TT_{1}-K_{1}\right)\lambda^{3}-Q\lambda^{2}-P\lambda -\Delta \Delta_{1},\\ & \lambda_{1,2}^{*}=\;\frac{-Q\pm \sqrt{Q^{2}-3\left(TT_{1}-K_{1}\right)P}}{3\left(TT_{1}-K_{1}\right)},\\ & p=-\frac{3F^{2}}{16E^{2}}+\frac{G}{2E},\;q=\frac{F^{3}}{32E^{3}}-\frac{FG}{8E^{2}}+\frac{H}{4E},\\ & \;w_{1,2}=\frac{-1\pm i\sqrt{3}}{2},\\ & D=\frac{q^{2}}{4}+\frac{p^{3}}{27},\;\;A_{j}={\left(-\frac{q}{2}\pm D^{1/2}\right)}^{1/3},\\ & x_{1}=A_{1}+A_{2},\;\;x_{2}=w_{1}A_{1}+w_{2}A_{2},\\ & x_{3}=w_{2}A_{1}+w_{1}A_{2},\;\;\Omega_{i}=x_{j}-\frac{F}{4E},\;i=1,2,3. \end{array}$$

**Theorem 6**

Let the following parameters be defined as in Theorem 5, then *Y*_*Q*_^*H*^(*iw*) < 0 for some *w* = *w*_0_ ∈ *R* if any one of the following conditions holds.

1. *a*_11_ > 0.
2. *a*_11_ = 0, *TT*_1_ − *K*_1_ > 0.
3. *a*_11_ = 0, *TT*_1_ − *K*_1_ = 0, *Q* > 0.
4. *a*_11_ = 0, *TT*_1_ − *K*_1_ = 0, *Q* < 0, ΔΔ_1_ > 0.
5. *a*_11_ = 0, *TT*_1_ − *K*_1_ = 0, *Q* < 0, *P* ≥ 0, ΔΔ_1_ − *P* ^2^/*Q*/4 > 0, ΔΔ_1_ ≤ 0,
6. *a*_11_ = 0, *TT*_1_ − *K*_1_ = 0, *Q* = 0, *P* > 0.
7. *a*_11_ = 0, *TT*_1_ − *K*_1_ = 0, *Q* = 0, *P* ≤ 0, ΔΔ_1_ > 0.
8. *a*_11_ = 0, *TT*_1_ − *K*_1_ < 0, ΔΔ_1_ > 0.
9. *a*_11_ = 0, *TT*_1_ − *K*_1_ < 0, ΔΔ_1_ ≤ 0, and *λ*_j_* ≥ 0, *h*(*λ*_j_*) < 0, for *j* = 1 or 2.
10. *a*_11_ < 0, *D* > 0, *Ω*_1_ > 0, *g*(*Ω*_1_) < 0.
11. *a*_11_ < 0, *D* < 0, and *Ω*_*j*_ ≥ 0, *g*(*Ω*_*j*_) < 0, for *j* = 1, 2 or 3.
12. *a*_11_ < 0, *D* = 0, *p* = *q* = 0, *g*(−*F*/4*E*) < 0.
13. *a*_11_ < 0, *D* = 0, *q* ^2^/4 = −*p* ^3^/27 ≠ 0, and *Ω*_*j*_ ≥ 0, *g*(*Ω*_*j*_) < 0, for *j* = 1 or 2.

**Proof**

YQH(iω)=Y¯Q(iω)+YQ(iω)=2Re[YQ(iω)], so *Y*_*Q*_(*iω*) to satisfy condition (2) in Lemma 2 equals to *Re*[*Y*_*Q*_(*iω*)] < 0,

(19) Re[YQ(iω)]=Re[iω−a11   −T1(iω)3+K1(iω)2+L1(iω)+Δ1(iω)4+T(iω)3+K(iω)2+L(iω)+Δ]=Eω8+Fω6+Gω4+Hω2+I(ω4−Kω2+Δ)2+(Lω−Tω2).

1. If *a*_11_ > 0, then *Re*[*Y*_*Q*_(*iω*)] < 0 when *ω* is large enough (See (1) of Theorem 6).
2. If *a*_11_ = 0, then
  (20) Re[YQ(iω)] =−(TT1−K1)ω6+Qω4+Pω2+ΔΔ1(ω4−Kω2+Δ)2+(Lω−Tω2).

Let *f*(*λ*) = −*Qλ* ^2^ − *Pλ* − ΔΔ_1_,

- If *TT*_1_ − *K*_1_ > 0, then *Re*[*Y*_*Q*_(*iω*)] < 0 when *ω* is large enough (See (2) of Theorem 6).
- If *TT*_1_ − *K*_1_ = 0, then
  - If *Q* > 0, then *Re*[*Y*_*Q*_(*iω*)] < 0 when *ω* is large enough (See (3) of Theorem 6).
  - If *Q* < 0,
    - If ΔΔ_1_ > 0, ∃ *ω*_0_ ∈ R, such that *Re*[*Y*_*Q*_(*iω*_0_)] < 0 (See (4) of Theorem 6).
    - If ΔΔ_1_ ≤ 0, solve *f*′(λ*) = 0, we can get λ* = −0.5*P*/*Q*, *f*(λ*) = 0.25*P* ^2^/*Q* − ΔΔ_1_.
    - Then when *P* ≥ 0, ΔΔ_1_ − 0.25*P* ^2^/*Q* > 0, ∃ *ω*_0_ > λ*, such that *Re*[*Y*_*Q*_(*iω*_0_)] < 0 (See (5) of Theorem 6).
  - If *Q* = 0, then *Re*[*Y*_*Q*_(*iω*)] = −*Pω* ^2^ − ΔΔ_1_.
    - If *P* > 0, then *Re*[*Y*_*Q*_(*iω*)] < 0 when *ω* is large enough (See (6) of Theorem 6).
    - If *P* ≤ 0, ΔΔ_1_ > 0, then ∃*ω*_0_, such that *Re*[*Y*_*Q*_(*iω*_0_)] = −ΔΔ_1_ < 0 (See (7) of Theorem 6).
- If *TT*_1_ − *K*_1_ < 0, let *h*(*λ*) = −(*TT*_1_ − *K*_1_)*λ* ^3^ − *Qλ* ^2^ − *Pλ* − ΔΔ_1_,
  - If ΔΔ_1_ > 0, then ∃*ω*_0_, such that *Re*[*Y*_*Q*_(*iω*_0_)] < 0 (See (8) of Theorem 6).
  - If ΔΔ_1_ ≤ 0, solve *h*(λ*) = 0, we can get
    (21) $$\lambda_{1,2}^{*}=\frac{-Q\pm \sqrt{Q^{2}-3\left(TT_{1}-K_{1}\right)P}}{3\left(TT_{1}-K_{1}\right)}.$$
    Then, for *i* = 1,2, if *λ*_i_* ≥ 0, *h*(*λ*_i_*) < 0, then ∃*ω*_0_, such that *Re*[*Y*_*Q*_(*iω*_0_)] < 0 (See (9) of Theorem 6).
- If *a*_11_ < 0, let *g*(*Q*) = *EQ* ^4^ + *FQ* ^3^ + *GQ* ^2^ + *HQ* + *I*, then *g*′(*Q*) = 4*EQ* ^3^ + 3*FQ* ^2^ + 2*GQ* + *H*. Let *x* = *Ω* + (*F*/4*E*), then the above becomes *g*′(*Q*) = 4*E*(*x* ^3^ + *px* + *q*) = 4*Ef*(*x*), then *x*_*i*_, *i* = 1,2, 3 are the roots of *f*(*x*) = 0, *Ω*_*i*_ are the roots of *g*′(*Ω*) = 0. If any one of the (10)–(13) of Theorem 6 holds, we can get *Re*[*Y*_*Q*_(*iω*_0_)] < 0.

So, if any one of conditions (1)–(13) holds, *Re*[*Y*_*Q*_(*iω*_0_)] < 0. *Y*_*Q*_(*s*) Satisfies condition (2) in Lemma 2. This completes the proof.

**Theorem 7**

For *j* = 1, or 2, let

(22) $$\begin{array}{c} w_{j}=\frac{\left(\sqrt{K+2\sqrt{\Delta }}+{\left(-1\right)}^{j}\sqrt{K-2\sqrt{\Delta }}\right)}{2},\\ A_{j}=L-3{w_{j}^{*}}^{2},\\ w_{j}^{*}=\sqrt{\frac{\left(K+{\left(-1\right)}^{j}\sqrt{K^{2}-4\Delta }\right)}{2}\;},\;\\ B_{j}=2Kw_{j}^{*}-4{w_{j}^{*}}^{3},\;\;A_{1j}=\Delta_{1}-K_{1}{w_{j}^{*}}^{2},\\ B_{1j}=L_{1}w_{j}^{*}-T_{1}{w_{j}^{*}}^{3}. \end{array}$$

Then *Y*_*Q*_(*s*) satisfies condition (3) of Lemma 2, if any one of the following conditions holds.

- $\Delta >0,\;K>2\sqrt{\Delta },\;T=L=0$, and any one of the following conditions holds.
  1. *K*_1_ *w*_1_^2^ − Δ_1_ ≠ 0.
  2. *K*_1_ *w*_1_^2^ − Δ_1_ = 0, (*L*_1_ − *T*_1_ *w*_1_^2^)(*w*_2_^2^ − *w*_1_^2^) > 0.
  3. *K*_1_ *w*_2_^2^ − Δ_1_ ≠ 0.
  4. *K*_1_ *w*_2_^2^ − Δ_1_ = 0, (*L*_1_ − *T*_1_ *w*_2_^2^)(*w*_1_^2^ − *w*_2_^2^) > 0.
- *K* > 0, Δ_1_ ≠ 0, Δ = 0, *L* = *KT* ≠ 0, and any one of the following conditions holds.
  1. *T*Δ_1_ < 0.
  2.   
*T*(*K* − *T*_1_ *K*) − Δ_1_ + *KK*_1_ ≠ 0.
  3.   
*T*(*K* − *T*_1_ *K*) − Δ_1_ + *KK*_1_ = 0, *T*(Δ_1_ − *KK*_1_) + *K*(*L*_1_ − *T*_1_ *K*) < 0.
-   Δ = 0, Δ_1_ *L* > 0.
- Δ < 0, or  *K* > 0, *K* ^2^ − 4Δ > 0, and $2L=T(K+\sqrt{K^{2}-4\Delta })(K+\sqrt{K^{2}-4\Delta }>0)$ or $2L=T\left(K-\sqrt{K^{2}-4\Delta }\right),\;(K-\sqrt{K^{2}-4\Delta }>0)$ and any one of the following conditions holds for *j* = 1, or 2.
  1. *A*_*j*_ *B*_1*j*_ − *A*_1*j*_ *B*_*j*_ ≠ 0.
  2.   
*A*_*j*_ *B*_1*j*_ − *A*_1*j*_ *B*_*j*_ = 0, *A*_*j*_ *A*_1*j*_ − *B*_*j*_ *B*_1*j*_ > 0.

**Proof**

Let *f*(*s*) = *T*_1_ *s* ^3^ + *K*_1_ *s* ^2^ + *L*_1_ *s* + Δ_1_, *g*(*s*) = *s* ^4^ + *Ts* ^3^ + *Ks* ^2^ + *Ls* + Δ, obviously, *∞* is not a single pole of *Y*_*Q*_(*s*) on the imaginary axis.

If *Y*_*Q*_(*s*) has a simple pole *s* = *iω* on the imaginary axis, where its associated residue

(23) $$k_{1}=\underset{s\to i\omega }{\lim}\left(s-i\omega \right)Y_{Q}\left(s\right)=\underset{s\to i\omega }{\lim}\left(s-i\omega \right)\frac{f\left(s\right)}{g\left(s\right)}={\frac{f\left(s\right)}{g^{\prime }\left(s\right)}|}_{s=i\omega }$$

is either a complex number or a negative real number, then *k*_1_ ≠ 0, so *f*(*s*) ≠ 0, which implies that *iω* is not a zero point of *f*(*s*) = 0, *iω*  is not a removed pole of *Y*_*Q*_(*s*).

(I) If *Y*_*Q*_(*s*) has four poles *s* = ±*iω*_1_, ±*iω*_2_  (*ω*_1_ ≠ *ω*_2_ ≠ 0) on the imaginary axis. In this case,  *g*(*s*) = (*s* ^2^ + *ω*_1_^2^)(*s* ^2^ + *ω*_2_^2^) = *s* ^4^ + (*ω*_1_^2^ + *ω*_2_^2^)*s* ^2^ + *ω*_1_^2^ *ω*_2_^2^. Hence we obtain *T* = *L* = 0, *K* = *ω*_1_^2^ + *ω*_2_^2^ > 0, Δ = *ω*_1_^2^ *ω*_2_^2^ > 0. Then, we can get $K+2\sqrt{\Delta }={\left(\omega_{1}+\omega_{2}\right)}^{2}$, $K-2\sqrt{\Delta }={\left(\omega_{1}-\omega_{2}\right)}^{2}$, which implies that $K>2\sqrt{\Delta }$, $\omega_{1,2}=(\sqrt{K+2\sqrt{\Delta }}\pm \sqrt{K-2\sqrt{\Delta }})/2$. So,
  (24) $$\begin{array}{c} \underset{s\to \pm i\omega_{1}}{\lim}\left(s\mp i\omega_{1}\right)Y_{Q}\left(s\right)=\frac{w_{1}\left(L_{1}-T_{1}w_{1}^{2}\right)\pm i\left(K_{1}w_{1}^{2}-\Delta_{1}\right)}{2w_{1}\left(w_{2}^{2}-w_{1}^{2}\right)}, \end{array}$$
  (25) $$\begin{array}{c} \underset{s\to \pm i\omega_{2}}{\lim}\left(s\mp i\omega_{2}\right)Y_{Q}\left(s\right)=\frac{w_{2}\left(L_{1}-T_{1}w_{2}^{2}\right)\pm i\left(K_{1}w_{2}^{2}-\Delta_{1}\right)}{2w_{2}\left(w_{1}^{2}-w_{2}^{2}\right)}. \end{array}$$
  Then, when condition (I) in Theorem 7 holds, *k*_1_ is a complex number or a negative real number. *Y*_*Q*_(*s*) satisfies condition (3) in Lemma 2.
(II) If *Y*_*Q*_(*s*) has a simple pole *s* = 0 and two conjugate poles ±*iω*  (*ω* ≠ 0) on the imaginary axis, and another pole is *a* ≠ 0.
In this case, it follows that Δ = 0, Δ_1_ ≠ 0, and *g*(*s*) has the form:
  (26) $$\begin{array}{cc} g\left(s\right) & =s\left({\text{s}}^{2}+\omega_{}^{2}\right)\left(\text{s}-a\right)\\ & ={\text{s}}^{4}-as^{3}+\omega_{}^{2}{\text{s}}^{2}-a\omega_{}^{2}, \end{array}\;$$
  which implies that *T* = −*a*, *K* = *ω* ^2^ > 0, *L* = −*aω* ^2^ = *KT*, Δ = 0, Δ_1_ = 0, Therefore,
  (27) $$g\left(s\right)=s\left({\text{s}}^{2}+K\right)\left(\text{s}+T\right)$$
  (1) The residue of *Y*_*Q*_(*s*) at *s* = 0 is
    (28) $$\underset{s\to 0}{\lim}sY_{Q}\left(s\right)=\frac{\Delta_{1}}{\left({\text{s}}^{2}+K\right)\left(\text{s}+T\right)}=\frac{\Delta_{1}}{KT}.$$
    Then, we conclude that if *K* > 0, Δ_1_ ≠ 0, Δ = 0, *L* = *KT* ≠ 0, *T*Δ_1_ < 0, *k*_1_ is a negative real number. *Y*_*Q*_(*s*) satisfies condition (3) in Lemma 2 (See (1) of (II) in Theorem 7).
  (2) The residue of *Y*_*Q*_(*s*) at $s=\pm i\sqrt{K}$ is

(29) $$\begin{array}{c} \underset{s\to \pm i\sqrt{K}}{\lim}\left(s\mp i\sqrt{K}\right)Y_{Q}\left(s\right)=\frac{T\left(\Delta_{1}-KK_{1}\right)+K\left(L_{1}-T_{1}K\right)\mp i\sqrt{K}\left(T\left(L_{1}-T_{1}K\right)-\Delta_{1}+KK_{1}\right)}{2K\left(K+T^{2}\right)} \end{array}.$$

Consequently, we conclude that if (2) or (3) in (II) in Theorem 7 holds, *k*_1_ is either an imaginary number or a negative real number. *Y*_*Q*_(*s*) satisfies condition (3) in Lemma 2.

(III) If *Y*_*Q*_(*s*) has a simple pole *s* = 0 on the imaginary axis, and the other poles are *a*_*i*_, *Re*[*a*_*i*_] ≠ 0, *i* = 1,2, 3, it follows that Δ_1_ ≠ 0, Δ = 0, and *g*(*s*) has the form
  (30) $$\begin{array}{cc} g\left(s\right) & =s\left(\text{s}-a_{1}\right)\left(\text{s}-a_{2}\right)\left(\text{s}-a_{3}\right)\\ & =s(s^{3}-\left(a_{1}+a_{2}+a_{3}\right)s^{2}\;\\ & \;\;+\left(a_{1}a_{2}+a_{1}a_{3}+a_{2}a_{3}\right)s-a_{1}a_{2}a_{3}). \end{array}\;$$
  Therefore we obtain that  Δ = 0, *T* = −(*a*_1_ + *a*_2_ + *a*_3_), *K* = *a*_1_ *a*_2_ + *a*_1_ *a*_3_ + *a*_2_ *a*_3_, *L* = −*a*_1_ *a*_2_ *a*_3_ ≠ 0, hence the reside of *Y*_*Q*_(*s*) at *s* = 0 is
  (31) $$\underset{s\to 0}{\lim}sY_{Q}\left(s\right)=\frac{\Delta_{1}}{a_{1}a_{2}a_{3}}=-\frac{\Delta_{1}}{L}.$$
  Then, when Δ = 0, Δ_1_ *L* > 0, *k*_1_ is a negative real number. *Y*_*Q*_(*s*) satisfies condition (3) in Lemma 2 (See (III) of Theorem 7).
(IV) If *Y*_*Q*_(*s*) has two conjugate poles ±*iω*(*ω* > 0) on the imaginary axis, and the other poles are *Re*[*a*] ≠ 0, *Re*[*b*] ≠ 0. In this case, *g*(*s*) has the form
  (32) $$\begin{array}{cc} g\left(s\right) & =\left(\text{s}-a\right)\left(\text{s}-b\right)\left(\text{s}+\omega^{2}\right)\\ & =s^{4}-\left(a+b\right)s^{3}+\left(ab+\omega^{2}\right)s^{2}\\ & \;-\left(a+b\right)\omega^{2}s+ab\omega^{2}. \end{array}\;$$
  Therefore, we obtain that *T* = −(*a* + *b*), *K* = *ab* + *ω* ^2^, *L* = −(*a* + *b*)*ω* ^2^, Δ = *ab* *ω* ^2^ ≠ 0. Then, *ab* = *K* − *ω* ^2^, Δ = (*K* − *ω* ^2^)*ω* ^2^⇔*ω* ^4^ − *Kω* ^2^ + Δ = 0. Solving it, we have
  (33) $$\omega_{j}^{*}=\sqrt{\frac{K\pm \sqrt{K^{2}-4\Delta }}{2}},\;j=1,2.$$
  It implies that Δ < 0 or *K* > 0, *K* ^2^ − 4Δ ≥ 0, and *T* = −(*a* + *b*) = *L*/*ω* ^2^. Then, the residue of *Y*_*Q*_(*s*) at *s* = ±*ω*_j_* is
  (34) $$\begin{array}{cc} & \underset{s\to \pm i\omega_{j}^{*}}{\lim}\left(s\mp i\omega_{j}^{*}\right)Y_{Q}\left(s\right)\\ & \;\;=\frac{-\left(A_{1j}A_{j}+B_{1j}B_{j}\right)\pm i\left(A_{1j}B_{j}-A_{j}B_{1j}\right)}{A_{j}^{2}+B_{j}^{2}}. \end{array}$$
  Hence, if condition (IV) in Theorem 7 holds, *k*_1_ < 0 or it is an imaginary number. *Y*_*Q*_(*s*) satisfies condition (3) in Lemma 2.

Therefore, when any one of conditions (I)–(IV) holds, *Y*_*Q*_(*s*) satisfies condition (3) in Lemma 2. This completes the proof.

**Theorem 8**

*Y*_*Q*_(*s*) has a multiple pole on the imaginary axis if any one of the following conditions holds.

- Δ = *L* = 0, Δ_1_ ≠ 0.
-   Δ = *L* = *K* = 0, and  Δ_1_ ≠ 0  or  *L*_1_ ≠ 0.
-   Δ = *L* = *K* = *T* = 0, and Δ_1_ ≠ 0 or *L*_1_ ≠ 0 or *K*_1_ ≠ 0.
- *T* = *L* = 0, *K* > 0, Δ = (*K*/2)^2^, and any one of the following conditions holds.
  1. 2Δ_1_ ≠ *KK*_1_.
  2.   2*L*_1_ ≠ *KT*_1_.

**Proof**

Let *f*(*s*) = *T*_1_ *s* ^3^ + *K*_1_ *s* ^2^ + *L*_1_ *s* + Δ_1_, *g*(*s*) = *s* ^4^ + *Ts* ^3^ + *Ks* ^2^ + *Ls* + Δ. Obviously, when the conditions (I)–(III) hold, 0 is the multiply poles of *Y*_*Q*_(*s*).

If *Y*_*Q*_(*s*) has two multiply nonzero poles ±*iω*  (*ω* > 0), then *g*(*s*) has the form:

(35) $$g\left(s\right)={\left(s^{2}+\omega^{2}\right)}^{2}=s^{4}+2\omega^{2}s^{2}+\omega^{4},$$

which implies that *T* = *L* = 0, *K* = 2*ω* ^2^ > 0, Δ = *ω* ^4^ = (*K*/2)^2^ > 0.

If ±*iω* are the multiply poles of *Y*_*Q*_(*s*), then*f*(*iω*) ≠ 0 where

(36) $$\begin{array}{cc} f\left(\pm i\omega \right) & =\left(\Delta_{1}-K_{1}\omega^{2}\right)\pm i\omega \left(L_{1}-T_{1}\omega^{2}\right)\\ & =\left(\Delta_{1}-\frac{KK_{1}}{2}\right)\pm i\omega \left(L_{1}-\frac{T_{1}K}{2}\right). \end{array}\;$$

Obviously, when any one of (1)-(2) of (IV) in Theorem 8 holds, *f*(*iω*) ≠ 0, any one of ±*iω* is a multiply pole of *Y*_*Q*_(*s*).

So, when any one of condition (I)–(IV) holds, *Y*_*Q*_(*s*) satisfies condition (4) in Lemma 2. This completes the proof.

When any one of Theorems 5–8 holds, *Y*_*Q*_(*s*) satisfies Lemma 2, which implies that the reaction-diffusion CNN with five state variables and one port at the equilibrium point is active.

These theorems can be implemented by a computer program for calculating the bifurcation diagram of the general corresponding CNN to determine emergence of complex dynamic patterns of the corresponding CNN.

## 4. Analysis and Simulations of Reaction-Diffusion CNN of HBV Mutation-Selection Model

Life systems consist of locally coupled homogeneous media. Mostly, dynamics of life systems are suitable to be described via locally connected reaction-diffusion CNNs. It may be expected that reaction-diffusion CNN will become a promising candidate for modeling life phenomena.

In Chapter 11 “Timing the emergence of resistance” (Page 110) of the book “Virus dynamic: mathematical principles of immunology and virology” (Oxford university press), Nowak et al. proposed a mathematical model which describes the mutation selection of HBV infection during the therapy [31]:

(37) $$\begin{array}{c} \frac{dx}{dt}=\lambda -dx-bvx-b_{n}xv_{n},\\ \frac{dy}{dt}=b\left(1-e\right)vx-ay,\\ \frac{dv}{dt}=ky-uv,\\ \frac{dy_{n}}{dt}=bevx+b_{n}xv_{n}-ay_{n},\\ \frac{dv_{n}}{dt}=k_{n}y_{n}-uv_{n}, \end{array}$$

where the five variables—*x*, *y*, *v*, *y*_*n*_, *v*_*n*_ represent the numbers of uninfected cells, infected cells infected by normal virus, normal virus, infected cells infected by mutated virus, and mutant viruses, respectively. *λ* is the rate of reproduction of uninfected cells. Uninfected cells die at rate *dx* and become infected at rate *bxv* by normal virus and infected at rate *b*_*n*_ *xv*_*n*_ by mutated virus. Infected cells infected by normal and mutated virus are removed at rate *ay* and *ay*_*n*_, respectively. Normal virus is produced at rate *ky* and removed at rate *uv*, mutated virus is produced at rate *ky*_*n*_ and removed at rate *uv*_*n*_. *e* is the rate constant describing the probability of mutation of virus (usual 10^−5^–10^−3^), *a*, *b*, *b*_*n*_, *d*, *e*, *k*, *k*_*n*_, *u*, *λ* are positive constants. The model was briefly analyzed in Nowak's book.

The reaction-diffusion CNN of HBV mutation selection of model has the form:

(38) $$\begin{array}{c} \frac{dx_{ij}}{dt}=\lambda -dx_{ij}-bx_{ij}v_{ij}-b_{n}x_{ij}v_{nij}+D_{1}\nabla^{2}x_{ij},\\ \frac{dy_{ij}}{dt}=b\left(1-e\right)x_{ij}v_{ij}-ay_{ij},\\ \frac{dv_{ij}}{dt}=ky_{ij}-uv_{ij},\\ \frac{dy_{nij}}{dt}=bex_{ij}v_{ij}+b_{n}x_{ij}v_{nij}-ay_{nij},\\ \frac{dv_{nij}}{dt}=k_{n}y_{nij}-{uv}_{nij}, \end{array}$$

where ∇^2^ *x*_*ij*_ = *x*_*i*−1*j*_ + *x*_*i*+1*j*_ + *x*_*ij*−1_ + *x*_*ij*+1_ − 4*x*_*ij*_.

Let equation (38) be zeros (*D*_1_ = 0) and solve it, we can get the two equilibrium points:

(39) $$\begin{array}{cc} Q_{1} & =\left(\frac{\lambda }{d},0,0,0,0\right), \end{array}$$

(40) $$\begin{array}{cc} Q_{2} & =(x_{0},\frac{u\left(au-b_{n}k_{n}ux_{0}\right)\left(\lambda -dx_{0}\right)}{bkx_{0}\left(b_{n}k_{n}ex_{0}+au-k_{n}x_{0}\right)},\;\\ & \;\;\frac{\left(au-b_{n}k_{n}ux_{0}\right)\left(\lambda -dx_{0}\right)}{bx_{0}\left(b_{n}k_{n}ex_{0}+au-k_{n}x_{0}\right)},\frac{eu\left(\lambda -dx_{0}\right)}{b_{n}k_{n}ex_{0}+au-k_{n}x_{0}},\\ & \;\;\frac{k_{n}e\left(\lambda -dx_{0}\right)}{b_{n}k_{n}ex_{0}+au-k_{n}x_{0}}), \end{array}$$

where *x*_0_ = *au*/((1 − *e*)*bk*) and *Q*_1_, *Q*_2_ stand for the patient's complete recovery and HBV persistent infection, respectively.

Consequently, the Jacobian matrix at the equilibrium point *Q*_*i*_  (*i* = 1,2) is

(41) $$J\left(Q_{i}\right)=\left[\begin{array}{ccccc} -d-bv-b_{n}v_{n} & 0 & -bx & 0 & -b_{n}x\\ b\left(1-e\right)v & -a & b\left(1-e\right)x & 0 & 0\\ 0 & k & -u & 0 & 0\\ bev+b_{n}v_{n} & 0 & bex & -a & b_{n}x\\ 0 & 0 & 0 & k_{n} & -u \end{array}\right].$$

**Figure 1:**
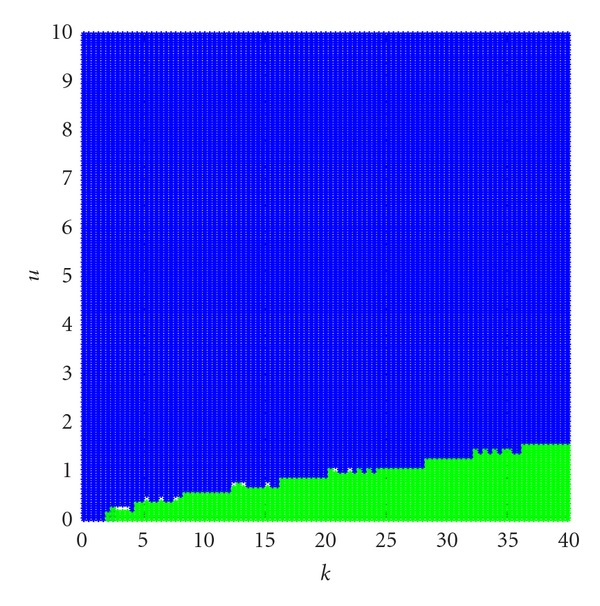
Bifurcation diagrams of equation (38) at the equilibrium points *Q*_1_ at *k* ∈ [0,40], *u* ∈ [0,10].

**Figure 2:**
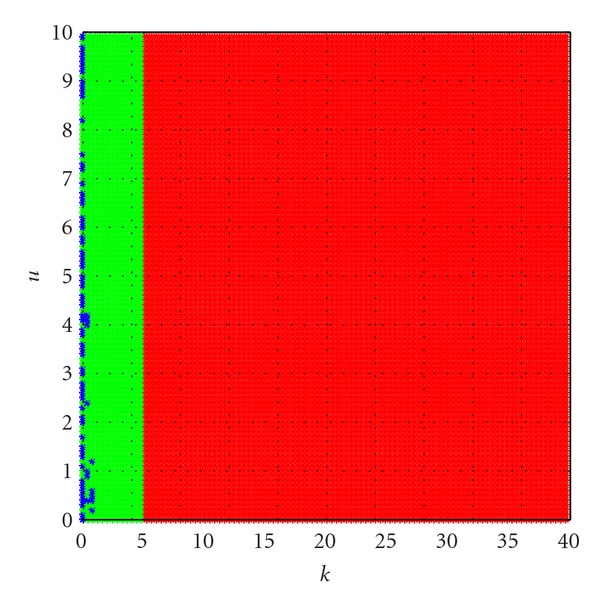
Bifurcation diagrams of equation (38) at the equilibrium points *Q*_2_ at *k* ∈ [0,40], *u* ∈ [0,10].

Taking *k*, *u* as variables, and *λ* = 10, *a* = 0.5, *b* = 0.01, *b*_*n*_ = 0.005, *e* = 0.0001, *k*_*n*_ = 10, and *d* = 0.01, using Theorems 5–8, we can calculate the bifurcation of the reaction-diffusion CNN model equation (38) at the equilibrium points *Q*_1_ and *Q*_2_ at *k* ∈ [0,40], *u* ∈ [0,10], see Figures 1 and 2.

In Figures 1 and 2, the domains are coded as follows: edge of chaos (locally active and stable) domain (shown red), locally active and unstable domain (shown green) and locally passive domain (shown blue). From Figure 1(a), we can see that the bifurcation at equilibrium point *Q*_1_ does not exist at the edge of chaos domain.

Take *λ* = 10, *k* = 0.01, *a* = 0.5, *b* = 0.01, *b*_*n*_ = 0.005, *k*_*n*_ = 10,  *e* = 0.0001, and  *k* = 1.0,3.0,4.9,5.1,10,24,39, *u* = 2,5, 9, we model the dynamic trajectories of equation (37) using MATLAB, see Table 1.

**Figure 3:**
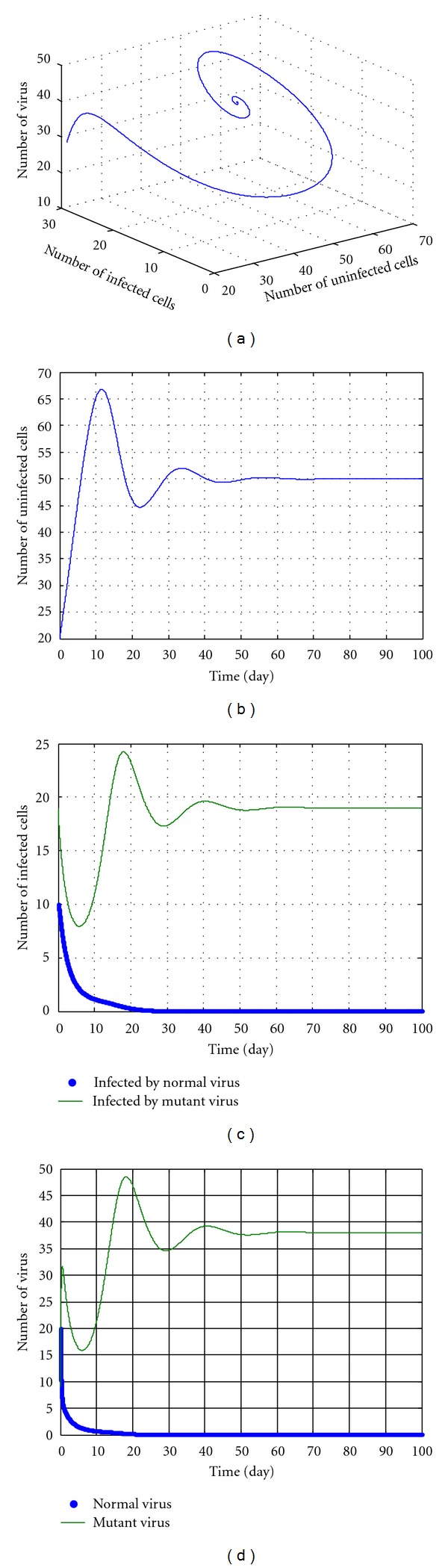
The kinetic trajectories of equation (37) when *u* = 5, *k* = 3.

**Figure 4:**
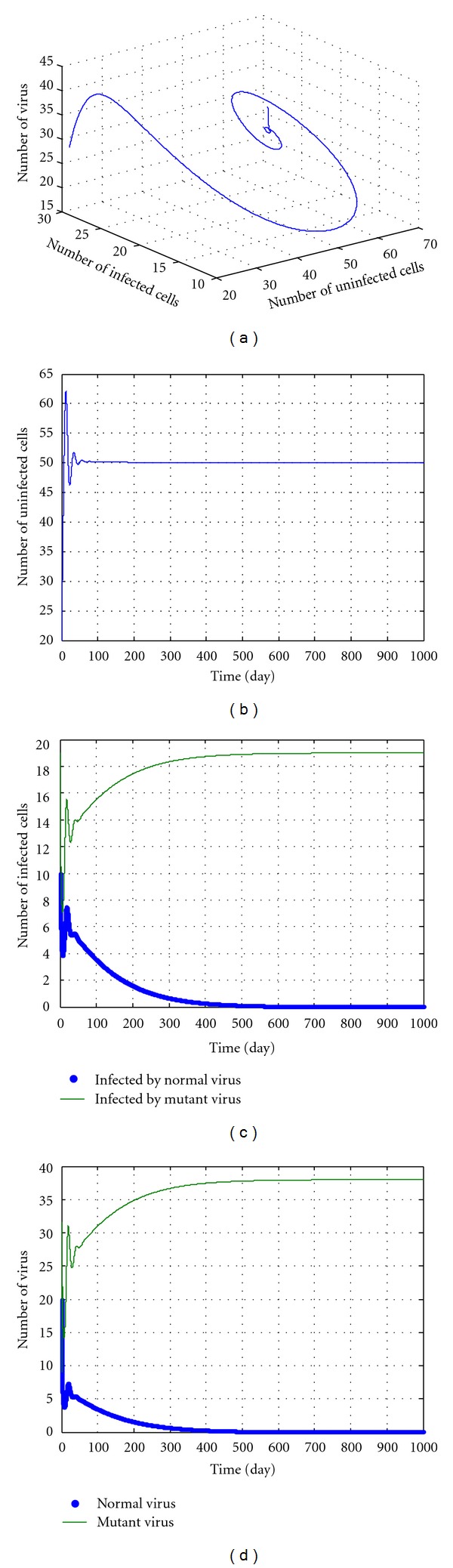
The kinetic trajectories of equation (37) when *u* = 5, *k* = 4.9.

**Figure 5:**
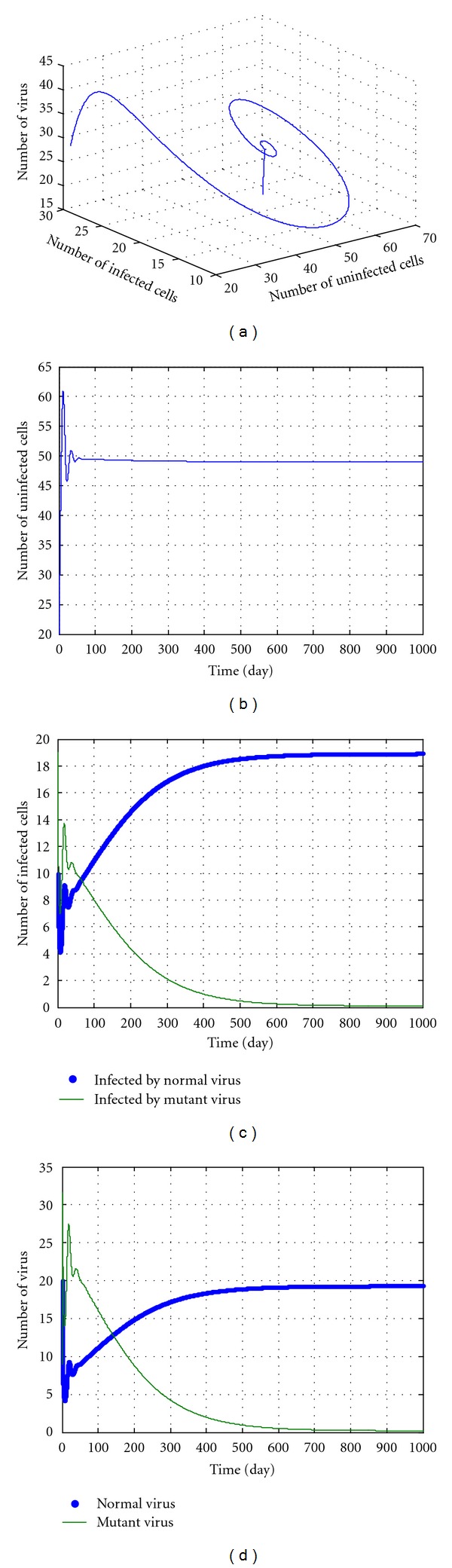
The kinetic trajectories of equation (37) when *u* = 5, *k* = 5.1.

**Figure 6:**
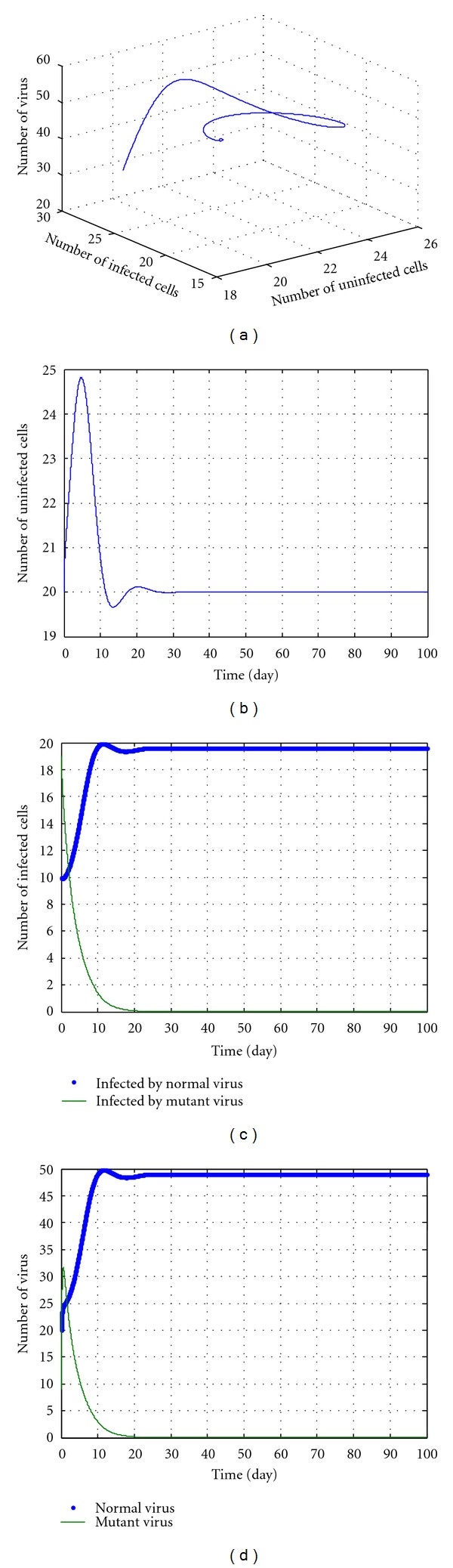
The kinetic trajectories of equation (37) when *u* = 5, *k* = 12.5.

**Figure 7:**
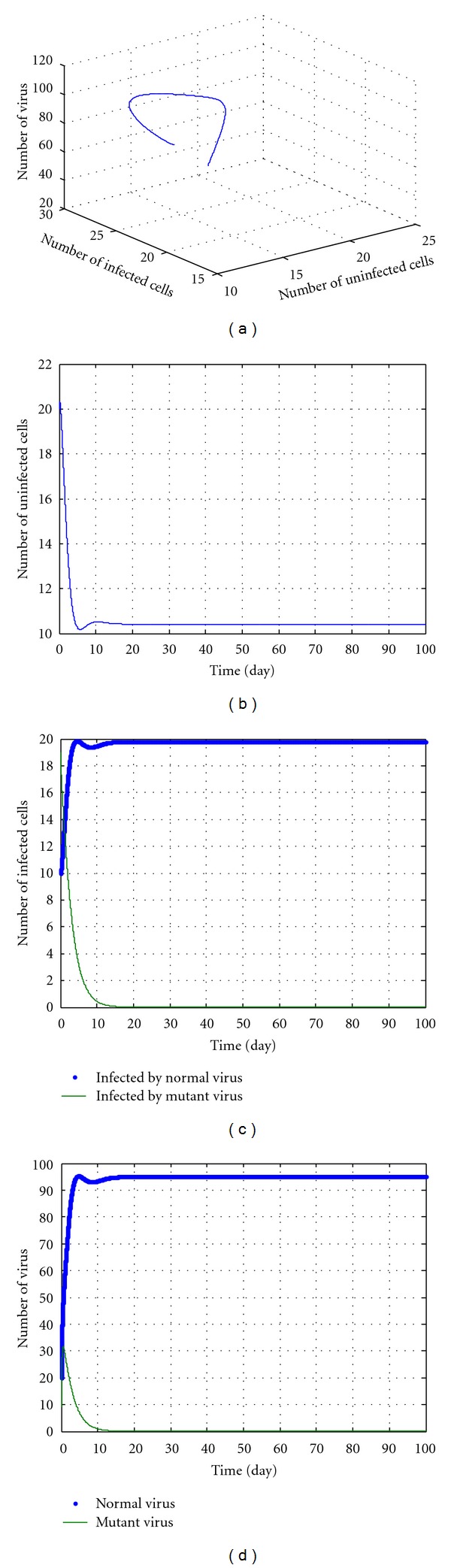
The kinetic trajectories of equation (37) when *u* = 5, *k* = 24.

In the following discussions, we select some parameters in No. 8-14 and *u* = 5, *k* = 12.5. The simulation results are shown in Figures 3, 4, 5, 6, and 7. During the simulation, we reached a new conclusion.

From Table 1 and Figures 3–7, we can conclude that

- when *k* is smaller (less than 5),
  1. these parameters are located in the green domain (the local and unstable domains);
  2. regardless of the value of *u*, the dynamic pattern of equation (37) is convergent or divergent depending on initial values;
  3. the No. 1, No. 4, and No. 5 variables in equation (37) increase and the No. 2 and No. 3 variables in equation (37) decrease to 0. This means the numbers of the mutant virus and of infected cells infected by mutant virus both increase, and the numbers of normal virus and of the infected cells infected by normal virus both decrease, even to near zero. Also, the No. 1 variable in equation (37), that is, the number of uninfected cells increases as compared to the initial number. All these indicate that the potency is perfect except for some virus mutation. The potency is ideal.
- When *k* is larger (greater than 5) but less than a threshold value (according to initial values and parameters, for example 12.5 in Figure 6), we can conclude the following.
  1. These parameters are located in the red domain (edge of chaos).
  2. Regardless of the value of *u*, the dynamic pattern of equation (37) is convergent.
  3. The No. 1, No. 2, and No. 3 variables in equation (37) increase and No. 4 and No. 5 variables decrease to 0. This means that the number of uninfected cells, the numbers of the normal virus, and of the cells infected by normal virus all increase. Meanwhile, the numbers of the mutant virus and of the cells infected by mutant virus both decrease, even to near zero. All these imply that the drug cannot clean the normal virus, but can destroy the mutant virus and increase the infection cells. The potency is also ideal.
- When *k* < 40 and greater than a threshold value (according to initial values and parameters),
  1. these parameters are located in the red domain (edge of chaos);
  2. regardless of the *u* value, the dynamic pattern of equation (37) is convergent;
  3. The No. 2 and No. 3 variables in equation (37) increase and No. 1, No. 4, and No. 5 variables decrease, which means the numbers of the mutant virus and of the uninfected cells decrease, and the number of normal virus increases. These imply that although the drug can prevent the mutation of the HBV effectively, it may destroy uninfected cells and the liver. The potency is not ideal.

## 5. Conclusions

The local activity of CNN has provided a powerful tool for studying the emergence of complex patterns in a homogeneous lattice formed by coupled cells. Based on the local activity principle, the analytic criteria for the local activity in reaction-diffusion CNN with five state variables and one port are set up. The analytical criteria include four theorems, which provide the inequalities involving the parameters of the CNN. The inequalities can be used for calculating the bifurcation diagram to determine emergence of complex dynamic patterns of the reaction-diffusion CNN. As an application example, a reaction-diffusion CNN of HBV mutation-selection model is analyzed and simulated, and the bifurcation diagrams are calculated. Numerical simulations show this CNN model may explain certain complex mutant conditions during the therapy. We conclude that the local activity theory provides a practical tool for the study of the complex dynamics of certain coupled nonlinear systems.

## Figures and Tables

**Table 1:** Cell parameters and correspongding dynamic properties of the reaction-diffusion CNN of HBV mutation-selection of HBV infection.

No.	*u*	*k*	Equilibrium point	Eigenvalues	Dynamic pattern
1	2	1.0	20,0,0,19,98	69.4396,29.8638, −0.0090, −33.3646, −71.9897	Convergent, divergent
2	2	3.0	20,0,0,20,98	−0.0097,53.0149,69.4400, −56.5150, −71.9902	Convergent, divergent
3	2	4.9	20,0,0,20,98	−0.0098,68.2343,69.4485, −71.6962, −72.0368	Convergent, divergent
4	2	5.1	20,20,50,0,0	−0.2043 ± 0.3878*i*, −0.0000, −2.6014, −2.5000	Convergent
5	2	10	10,20,99,0,0	−2.7130, −0.3935 ± 0.4583*i*, −0.2192, −2.2808	Convergent
6	2	24	4,20,239,0,0	−3.2812, −0.8094 ± 0.2709*i*, −0.3768, −2.1232	Convergent
7	2	39	3,20,389,0,0	−4.4393, −1.2723, −0.6884, −0.4059, −2.0941	Convergent
8	5	1.0	50,0,0,19,38	67.9709,28.4103, −0.0075, −34.9127, −73.5210	Convergent, divergent
9	5	3.0	50,0,0,19,38	−0.0092,51.5420,67.9713, −58.0426, −73.5215	Convergent, divergent
10	5	4.9	50,0,0,19,38	−0.0095,66.7552,67.9801, −73.2207, −73.5651	Convergent, divergent
11	5	5.1	49,19,19,0,0	−0.0920 ± 0.2787*i*, −0.0092, −5.5160, −5.4909	Convergent
12	5	10	25,19,39,0,0	−0.1829 ± 0.3778*i*, −0.2375, −5.5343, −5.2625	Convergent
13	5	24	10,20,95,0,0	−5.5708, −0.4446 ± 0.4784*i*, −0.3915, −5.1085	Convergent
14	5	39	6,20,155,0,0	−5.6298, −0.7151 ± 0.4210*i*, −0.4343, −5.0657	Convergent
15	9	1.0	90,0,0,18,20	66.0620,26.5823, −0.0055, −37.0867, −75.6121	Convergent, divergent
16	9	3.0	90,0,0,18,20	−0.0085,49.6418,66.0624, −60.1432, −75.6126	Convergent, divergent
17	9	4.9	90,0,0,18,20	−0.0091,64.8330,66.0717, −75.3029, −75.6527	Convergent, divergent
18	9	5.1	88,18,10,0,0	−0.0531 ± 0.2110*i*, −0.0106, −9.5037, −9.4894	Convergent
19	9	10	45,19,21,0,0	−0.1047 ± 0.2973*i*, −0.2431, −9.5106, −9.2569	Convergent
20	9	24	19,20,52,0,0	−0.2481 ± 0.4288*i*, −9.5339, −0.3897, −9.1103	Convergent
21	9	39	12,20,86,0,0	−0.4014 ± 0.4931*i*, −9.5671, −0.4300, −9.0700	Convergent
